# Letter to the Editor on “Is remote blood pressure monitoring and management a better approach for patients with hypertension? A narrative review”

**DOI:** 10.1111/jch.14704

**Published:** 2023-07-21

**Authors:** Amirali Ahrabi

**Affiliations:** ^1^ School of Medicine Tehran University of Medical Sciences Tehran Iran; ^2^ School of Public Health Tehran University of Medical Sciences Tehran Iran

Dear Editor,

I read with great interest the well‐written and well‐made review by Li and colleagues. recently published in the “Journal of Clinical Hypertension^1^” assessing advantages and disadvantages of remote blood pressure (BP) monitoring as well as its efficacy.

There are various digital health innovations available for hypertension management, such as cuffless BP sensors, wireless smartphone‐enabled BP monitors for the upper arm, mobile applications, and remote monitoring technologies. These technological advancements can be beneficial for confirming hypertension before treatment, particularly with the growing focus on home and ambulatory BP monitoring. Nevertheless, there are issues related to privacy, accuracy, and cost that have impeded their widespread adoption in clinical settings.^2^

Abdullah and colleagues highlighted the importance of patients' acceptance of telemonitoring of home BP and reported that patients found the BP telemonitoring service user‐friendly, but faced challenges in finding it beneficial. Patients expressed difficulties in interpreting their monitored home BP readings and desired more feedback from their doctors. To improve user satisfaction, they proposed making enhancements to the telemonitoring capabilities of the BP system.^3^

Similar studies show that for optimizing investment in remote telemonitoring, special attention should be paid to identify groups of patients who are more probable to benefit from the intervention^4^ and also the individual needs of each target group must be analyzed at the beginning, particularly if elderly and poorly educated patients are involved to ensure full and easy access to technology‐based telemonitoring for their own healthcare.^5^

To make telemedicine interventions accessible and affordable in countries with limited resources and infrastructure, a collaborative and policy‐making approach is essential. This involves working together among various organizations, including health‐care organizations, the Ministry of Communications, insurance companies, and banks. Through this approach, telemedicine services can be made available to everyone, regardless of their location or socio‐economic status, which can help improve the overall quality of health care and public health.^6^, ^7^

More clinical trials are needed to prove the safety and effectiveness of information and communication technology‐based care for hypertension. However, implementing telemonitoring and telemedicine in clinical practice is essential to address the hypertension paradox. Despite challenges related to cost, data integration, team‐based care redesign, and user experience, information and communication technology‐based hypertension management is likely to play a vital role in improving public health.^8^

## AUTHOR CONTRIBUTIONS

Amirali Ahrabi is the sole contributor and takes full responsibility for its content. He conducted thorough research, reviewed relevant literature, consulted with experts to develop a comprehensive understanding of the issue at hand. The author wrote the letter independently and took care to ensure the writing was clear, concise, and persuasive. The author believes that open and honest dialogue is critical to addressing complex issues facing society and hopes that his letter contributes to this dialogue in a meaningful way.
